# Facemask Usage Among Pedestrians in Most Crowded Urban Districts of Kabul, Afghanistan, During the Third COVID-19 Wave: An Observational Study

**DOI:** 10.4269/ajtmh.21-1070

**Published:** 2022-04-11

**Authors:** Arash Nemat, Abdullah Asady, Ahmad Wali Ataye, Tamim Jan Danishmand, Mohammad Yasir Essar, Nahid Raufi

**Affiliations:** ^1^ Department of Microbiology, Kabul University of Medical Sciences, Kabul, Afghanistan;; ^2^ Department of Cardiology, Nanfang Hospital, Southern Medical University, Guangzhou, China;; ^3^ Department of Surveillance, Ministry of Public Health, Kabul, Afghanistan;; ^4^ Kabul University of Medical Sciences, Kabul, Afghanistan;; ^5^ Department of Dermatology, Maiwand Hospital, Kabul University of Medical Sciences, Kabul, Afghanistan

## Abstract

COVID-19 has spread worldwide since its emergence from Wuhan. In countries with a low vaccination rate, the use of facemasks is essential to limit the risk of COVID-19 transmission. We have conducted this study in June 2021 to estimate the prevalence of facemask usage, and investigate the use of different types of facemasks and their distribution among pedestrians in the most crowded urban districts of Kabul during the third COVID-19 wave in Afghanistan. Using a multistage sampling method, a total of 5,000 pedestrians were selected from five most crowded urban districts of the city. The data was gathered by an observational method. The percentage, mean, and standard deviation were used to describe the variables. The χ^2^ test analysis was used to assess the relationship between two categorical variables. Of the 5,000 observations, the most common age group was 10–39 years with high participation of male (87.2%). A total of 2,013 (40.3%) people used facemasks (95% CI). Females used facemasks significantly more than males (64.6% versus 36.7%, *P* < 0.001). Among the pedestrians who used a facemask, most of them (88.6%) wore their facemask correctly. In conclusion, the prevalence of facemask use in Kabul was fairly low especially among elderly people (≥ 60 years). Hence, the observed rates probably cannot protect the community against COVID-19. Therefore, it is important to emphasize the public health recommendations via educational programs and national campaigns to support the strict use of facemasks in public places.

## INTRODUCTION

COVID-19 first emerged in Wuhan, China, and soon became a global threat.^1^ This virus spreads via respiratory droplets and direct contact, and has caused significant damage, both to health and economics.^2^ The first confirmed case of COVID-19 in Afghanistan was reported on February 24, 2020,^3^ and then the disease spread rapidly to most areas of the country including Kabul, which is assumed to be the most densely populated city of the country.^4^ Although the actual figures of the infection rates seem different from that of official reports, still the 132,777 confirmed cases with 5,638 death would be remarkable.^5^^,^^6^

Lower access to the COVID-19 vaccine in many countries necessitates the people to follow preventive measures until the vaccination process covers the entire population.^7^ As of February 9, 2022, a new wave of COVID-19 struck the healthcare system.^8^ The strategies to combat the disease are mainly focused on social distancing and the use of facemask.^9^ The Ministry of Public Health (MOPH) of Afghanistan reported, on June 10, 2021, that 483,580 Afghans have received the first dose of the COVID-19 vaccine and only 171,693 individuals (around 0.5% of Afghanistan population) had received two doses. Among vaccine recipients, only 68,152 (40%) persons were above 50 years old.^10^ Moreover, the withdrawal of the United States and international allies have presented the healthcare system with more challenges. Over the years, Afghanistan health system was operating through external aids and funding. The withdrawal has caused the suspension of funding. As a result, Afghanistan healthcare system has stopped functioning properly. Since then, the COVID-19 pandemic numbers are uncertain in the country. There is a lack of a surveillance system to report the daily cases. In addition to this, some COVID-19 hospitals have stopped functioning due to a lack of staff and funding.^11^

In a country like Afghanistan where insufficient doses of vaccines are available to the public, it is crucial that people strictly follow recommendations of WHO regarding facemask usage.^12^ Studies suggest that people aged 2 years or more are required to use facemasks in crowded places for prevention of possible transmission.^13^ Meanwhile, physical distancing, avoiding crowds, cleaning hands, and coughing into a bent elbow or tissue and living in well-ventilated rooms are the main advices of WHO for prevention of the infection.^14^ To increase the effectiveness, appropriate use of facemask is essential. In other words, the mask should cover nose and mouth, have two or more layers of washable and breathable fabric, and be used with clean hands.^14^

Although the MOPH Afghanistan advice is in-line with WHO framed recommendations on the use of facemasks and their proper usage in the community, the extent of COVID-19 cases in the country indicates that the MOPH should take more serious actions to encourage, and support adherence to reduce the transmission rates.^15^

### Objective.

This study aimed to examine the prevalence of facemask usage among pedestrians in Kabul to determine peoples’ behaviors of the preventive measures and provide additional insight to the national and international healthcare organizations regarding the situation.

## MATERIALS AND METHODS

### Study design.

This population-based study was conducted in Kabul, Afghanistan, during June 10–20, 2021, with the primary objective of assessing the usage of facemasks among pedestrians through nonparticipant observation. A total of 5,000 passers-by were observed in five districts of the city. Data was collected through observation of passers-by in the streets. This method was chosen over self-reported survey as it has been identified as a more reliable measure of adherence.^16^

### Inclusion and exclusion criteria.

All pedestrians who were 2 years old or more were included in the study. Exclusion criteria for the study were: 1) a passer-by whose face was fully covered and the observer was not able to detect whether he/she wore a mask (women may wear veils); and 2) short-term appearance of a walker to the observer so that he/she was not able to conclude whether the subject used a facemask.

### Data collection.

For data collection, eight observers were used who had bachelor degrees in Health and Behavioral Sciences. Before starting the observation, a 2-hour training session was conducted by the principal investigator to instruct observers on the methods of observation, subject selection, and completion of the checklist. To ensure the quality of data collection, two expert supervisors accompanied the observers during the first 2 days. Observers’ performance was continuously monitored by the assigned supervisors. Checklists filled out by observers were reviewed by supervisors and appropriate feedbacks were given.

Observation points were determined on the basis of a detailed map of Kabul city and the most crowded places of the city were selected. At each point, gender, approximate age, usage of facemask, gloves and shield, type of facemask, and correct use of facemask were recorded. Insufficient coverage of the mouth and nose, wearing facemask upside down or inside-out were defined as “incorrect or unacceptable” ways of facemask usage. The observers were placed at the observation stations from where they could closely watch the types of facemasks. If a mask was not distinguishable, the observers did not include in the study. Surgical masks were observed as face masks with their specific manner that cover the nose and month without any filtered nope. In Afghanistan, these masks are usually in light blue color that was easily differentiated from a filtered N95 mask that are commonly used in healthcare settings and are a subset of N95 Filtering Facepiece Respirators (FFRs). Observation was performed during the busy hours of each area from 6.00 am to 10.00 am and 3.00 pm to 7.00 pm every day.

### Sample size and sampling method.

To determine the minimum sample size, we used the formula for estimating a population proportion. For this purpose, α  =  0.05, *p*  =  0.5, d  =  0.04, and a design effect of 1.6 were considered. A minimum sample size of 1,000 estimated for each district, thus, the final sample size needed for this study was 5,000 subjects. We used a multistage sampling method. Each of the five urban districts was considered as a stratum. Then, the defined number of clusters were assigned to each neighborhood. Each cluster consisted of 100 pedestrians. The location of the observation stations was determined by a targeted sampling strategy from the busy and most crowded passages of each neighborhood. Meanwhile, to prevent the Hawthorne effect, the observation stations were chosen in the places where the subjects were not able to notice the observers, whereas the observers were able to closely watch the subjects and coordinated to record their findings.

A nonprobability convenience sampling method was used in the last stage. At each observation station, the closest pedestrian to the observer was selected as the first sample and included in the study. Then, the next closest person to the observer selected as the next sample and this was continued until the total number of selected persons in each cluster reached 50 in the morning and 50 in the evening. Finally, a total of 50 clusters of 100 people were assessed from five crowded places in the city.

### Statistics.

Statistical Package for Social Studies v. 25 used for analysis. Descriptive statistical measures including mean, standard deviation and percentage used to describe the data. The estimated prevalence rates presented with 95% confidence interval. χ^2^ analyses used to assess relationship between categorical variables. A *P* value of < 0.05 considered statistically significant at 95% confidence interval.

## RESULTS

A total of 5,000 pedestrians were assessed to observe their adherence with facemask usage. Among the observed individuals, 4,359 (87.2%) were male. Three quarters of the subjects included in the 10–39 years age group. Almost 40% of the people used facemasks. The most common type of facemask used by the observed pedestrians was the surgical (medical) masks (89.6%). Gloves and shields were used by only 0.4% and 0.3% of subjects, respectively. Demographic characteristics and frequency of personal protective measures are shown in Table 1.

**Table 1:** Demographic characteristic and frequency of personal protective measures among pedestrians in Kabul [*N* = 5,000]

Variable		*N*	%
Age (years)			
2–9	–	160	3.2
10–39	–	3,756	75.1
40–59	–	898	18.0
≥ 60	–	186	3.7
Sex			
Male	–	4,359	87.2
Female	–	641	12.8
Facemask usage			
Yes	–	2,013	40.3
No	–	2,987	59.7
Type of mask [*N* = 2,013]			
Surgical	–	1,804	89.6
Cloth	–	139	6.9
Filtered	–	52	2.6
Other	–	18	0.9
Way of face mask usage [*N* = 2,013]			
Correct	–	1,779	88.3
Uncovered mouth and/or nose	–	157	7.8
Inside-out	–	56	2.8
Upside-down	–	22	1.1
Gloves usage			
Yes	–	22	0.4
No	–	4,978	99.6
Shield usage			
Yes	–	17	0.3
No	–	4,983	99.7
District			
First district (Deh Afghanan)	–	1,100	22.0
11th district (Saray-e-Shamali)	–	950	19.0
10th district (Airport)	–	800	16.0
Second district (Sar-e-Chawk)	–	1,150	23.0
Third district (Kote Sangi)	–	1,000	20.0
Time			
am	–	2,250	45.0
pm	–	2,750	55.0

Table 2 shows the frequency of facemask usage among various groups of subjects. The highest prevalence of facemask usage was seen in the age group of 40–59 years, and the lowest was found in the age group of 2–9 years. Furthermore, almost 40% of the subjects aged 60 years and over were more likely to use facemasks (Table 2).

**Table 2:** Prevalence rates of face mask usage by sex, age group, and districts

	Number of observed pedestrians	Face mask usage	
		*N*	(CI 95%) prevalence
Overall prevalence	5,000	2,013	–
Sex			
Male	4,359	1,599	36.7%
Female	641	414	64.6%
Age group			
2–9 years	160	24	15.0%
10–39 years	3,756	1,450	38.6%
40–59 years	898	463	51.6%
60 or more	186	76	40.9%
District			
First district	1,100	458	41.6%
Second district	1,150	393	34.2%
Third district	1,000	463	46.3%
10th district	800	384	48.0%
11th district	950	315	33.2%

Generally, the prevalence of facemask usage increased significantly by age (*P* for trend < 0.001), except for those aged 60 years or more who used facemasks less often than the age group 40–59 years. The prevalence of facemask among females was significantly higher than that of males (64.6% versus 37.7%, *P* < 0.001).

**Figure 1.**
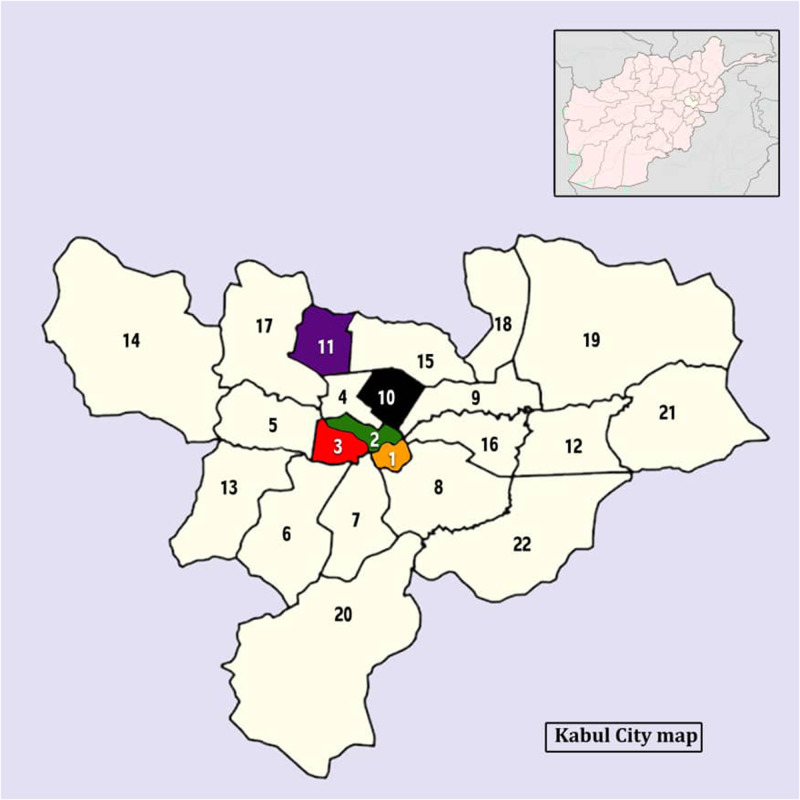
Kabul urban districts map. This figure appears in color at www.ajtmh.org.

The prevalence of facemask usage among pedestrians of the five districts of Kabul city varied. Overall, the prevalence of facemask usage was below 50% in all observed districts. The highest prevalence of facemask usage observed in 10th district (48%) whereas the lowest prevalence found in 11th district (33.2%) (Figure 1).

The colored areas indicate districts 1, 2, 3, 10, and 11 where the study took place.

Among the pedestrians who used a facemask, surgical masks were used widely by male and female (90.6% and 86%, respectively). The prevalence of using cloth masks was 6.9% in all subjects, with females two times more likely than males (12.8% versus 5.4%, *P* < 0.001). In addition, the filtered masks were used in a lower rate (2.6%), with male subjects using two times more likely than females to use them (2.9% versus 1.2%, *P* < 0.001).

The majority (88.3%) of the observed pedestrians wore facemasks correctly, whereas 7.8% did not cover their mouths and noses completely, 2.8% wore them inside-out, and 1.1% wore them upside-down. There was no statistically significant difference between men and women in correct way of wearing of facemasks (*P* = 0.227). In addition, 0.4% and 0.3% of pedestrians wore gloves and shields respectively with no statistically significant difference between the sex groups (*P* = 0.451 and *P* = 0.896) (Table 3).

**Table 3:** Association between personal protective measures and sex among pedestrians in Kabul

Variables	Male *N* (%)	Female *N* (%)	Total *N* (%)	*P* value*
Types of face mask				0.0001
Surgical mask	1,448 (90.6)	356 (86.0)	1,804 (89.6)	
Cloth mask	86 (5.4)	53 (12.8)	139 (6.9)	
Filtered mask	47 (2.9)	5 (1.2)	52 (2.6)	
Other	18 (1.1)	0 (0.0)	18 (0.9)	
Total	1,599 (100)	414 (100)	2,013 (100)	
How to use facemask				0.227
Correct use	1,406 (87.9)	372 (89.9)	1,778 (88.3)	
Uncovered mouth and/or nose	134 (8.4)	23 (5.6)	157 (7.8)	
Inside-out	43 (2.7)	13 (3.1)	56 (2.8)	
Upside-down	16 (1.0)	6 (1.4)	22 (1.1)	
Total	1,599 (100)	414 (100)	2,013 (100)	
Gloves use				0.451
Yes	18 (0.4)	4 (0.6)	22 (0.4)	
No	4,341 (99.6)	637 (99.4)	4,978 (99.6)	
Total	4,359 (100)	641 (100)	5,000 (100)	
Shield use				0.896
Yes	15 (0.3)	2 (0.3)	17 (0.3)	
No	4,344 (99.7)	639 (99.7)	4,983 (99.7)	
Total	4,359 (100)	641 (100)	5,000 (100)	

*Indicates statistical significance.

## DISCUSSION

Afghanistan entered into the third wave of the COVID-19 pandemic in May 2021.^17^ Prevention measures are effective means of reducing disease transmission, especially when there is insufficient supply of vaccines, as reported from Afghanistan.^17^ In such conditions, it is important to know the public’s adherence to prevention and protection measures, such as using facemask, social distancing, and avoiding crowded areas to protect ourselves and society from the virus. The aim of this study was to observe the usage of facemasks among pedestrians of five districts of Kabul City, Afghanistan.

The overall prevalence of facemasks among pedestrians was 40.3%. This finding is similar to reports of studies conducted among pedestrians in Ahvaz of Iran (45.6%) and Wisconsin (41.5%).^18^^,^^19^ However, this figure is lower than the findings from Japan (80.9%), Northern Vermont (75.5%), and Nigeria (65.5%).^20^^–^^22^ This level of facemask usage does not seem to reduce COVID-19 transmission effectively. A study conducted by Tian et al. (2021) on February 2021 estimated that if 60% of population wear facemasks with 60% efficiency in filtering aspired air, the basic reproduction number of COVID-19 would reach one and the pandemic would be controlled.^23^

There was a higher prevalence of facemask wearing among adults aged 40–59 years compared with those aged 60 years or more. This finding contrasts with the patterns reported in other studies.^19^^,^^24^ This might be due to the coverage of vaccination among elderly people who might feel safer than others. Moreover, illiteracy and listening to conspiracy theories may also have contributed to negligence of facemask wearing by elderly people. This is a risky practice that may increase the disease transmission both among this vulnerable group and the society at large. Further, the pandemic and conflict have caused significant economic burden on Afghanistan. Poor people are not able to buy masks to protect themselves that cost 10 AFG (0.1 USD). With the current economic crisis, the depth of poverty has massively escalated. People cannot afford to stay at home and not work. They need to shop daily as supplies are variable and so need to travel into congested places often without a mask.^25^^,^^26^

Female subjects were more likely to adhere to precautionary measures, including the usage of facemask (64.6%), compared with male individuals (36.7%). This finding is similar to a study conducted in Northern Vermont and Southwest Iran.^16^^,^^22^ Women are generally reported to pay more attention to their health and wellbeing.^16^

Among five urban districts of Kabul city, the prevalence of facemask usage was significantly higher among pedestrians of 10th district near to the Kabul international airport (48%, *P* < 0.001). It is possible that among the observed people, there might have been those who had flights for which mask wearing was compulsory or people may perceive themselves at higher risk near an airport.

There are different types of facemasks available such as homemade facemasks, surgical masks, and respirators. Homemade facemasks may be used by public and health personnel in accordance with CDC recommendations. Surgical masks are considered mainly for medical personnel. They cover nose and mouth and provide a physical barrier against fluid/droplets and particulate materials. Although these masks are basically used for medical purposes, they cannot provide full protection against germs and a loose fit between the surface of the mask and the face increases the risk. Respirators, known as FFRs tightly adhere to face and provide certain filtration efficiency levels to help reduce exposure to pathogenic airborne particles in a healthcare setting.^27^

In terms of how to use facemasks, CDC has provided several recommendations; facemasks should cover nose and mouth and must be secured under the chin; facemasks should fit snugly against the side of face; when removing a facemask, carefully untie the strings behind the head; handle only by ear loops; fold the outside corners together; wash your hands immediately after removing the facemask, and do not touch your eyes and mouth while removing the mask.^28^

In Afghanistan, educational and awareness programs should be conducted in local languages (Dari, Pashto) for educating people on how to use facemasks. The majority of the population are illiterate. Awareness programs would help them understand the importance of using facemask. Moreover, access and affordability to facemasks should be improved to address this barrier to proper use. This calls for collaboration between the government, nonprofit organizations, and the international community

## STRENGTHS AND LIMITATIONS

Our study has a number of strengths and limitations. First, this is the first study conducted in Afghanistan evaluating the usage of facemask among pedestrians in most crowded urban districts of Kabul. Second, our article evaluates the adherence of pedestrians to facemask through observation without any intervention. The main weakness of the article is that our observers could not observe those individuals who were unemployed or stayed at home all the time. Our study focused merely on individuals attending their jobs or other activities daily.

## CONCLUSION

The prevalence of facemask usage in Kabul was fairly low particularly among the vulnerable group of ≥60 years old. Hence, community protection against COVID-19 requires more efforts and attention. Therefore, it is important to emphasize public health recommendations via educational programs and national campaigns to convince people to wear facemasks in public places, and increase access to the means of adhering to these recommendations.
